# Molecular marker sequences of cattle *Cooperia* species identify *Cooperia spatulata* as a morphotype of *Cooperia punctata*

**DOI:** 10.1371/journal.pone.0200390

**Published:** 2018-07-06

**Authors:** Sabrina Ramünke, Fernando de Almeida Borges, Elke von Son-de Fernex, Georg von Samson-Himmelstjerna, Jürgen Krücken

**Affiliations:** 1 Institute for Parasitology and Tropical Veterinary Medicine, Freie Universität Berlin, Berlin, Germany; 2 Universidade Federal do Mato Grosso do Sul (UFMS), Campo Grande, Brazil; 3 Centro de Enseñanza Investigación y Extensión en Ganadería Tropical, Facultad de Medicina Veterinaria y Zootecnia, Universidad Nacional Autónoma de México, H. Tlapacoyan, Veracruz, México; National Cheng Kung University, TAIWAN

## Abstract

The genus *Cooperia* includes important parasites of ruminants and currently contains 34 accepted species. However, even for those species infecting livestock, there is a considerable lack of molecular information and many species are only identifiable using subtle morphological traits. The present study aimed to provide molecular data to allow diagnosis of *Cooperia* species infecting cattle. Partial sequences of two mitochondrial (cytochrome oxidase 2, 12S rRNA gene) and two nuclear genes (isotype 1 β tubulin gene including two introns, internal transcribed spacers (ITS) were obtained from morphologically identified specimens of *Cooperia pectinata*, *Cooperia punctata* and *Cooperia spatulata* as well as from larvae of pure *Cooperia oncophora* and *C*. *punctata* laboratory isolates. Pairwise identity of ITS-2 sequences was very high and it was the only region able to identify a specimen as *Cooperia* sp. However, the ITS-2 was unreliable for diagnosis at the species level. All other marker sequences could not unequivocally be allocated to the genus *Cooperia* but allowed clear species identification with the exception of the pair *C*. *punctata*/*C*. *spatulata* for which no significant differences were found for any marker sequence. Maximum-likelihood phylogenetic analyses of individual genes as well as a multi-locus analysis covering all four sequences confirmed that specimen identified as *C*. *spatulata* were randomly distributed throughout the *C*. *punctata* cluster and formed no group of their own. In contrast, the other *Cooperia* species formed clearly separated and statistically supported clusters. These data indicate that *C*. *spatulata* is most likely only a morphotype of *C*. *punctata* and the name should be considered a synonym. Combinations of nuclear and mitochondrial markers should be used to identify morphotypes or cryptic species to benefit from excellent barcoding properties of the latter but allowing proper phylogenetic analyses and controlling for lineage sorting that might occur for mitochondrial genotypes within a species.

## Introduction

*Cooperia*, Ransom, 1907 is a genus of gastrointestinal nematodes (GINs) that belongs to the superfamily Trichostrongyloidea [1]. Species in the genus parasitize in the small intestine of ruminants and several species affect both wild and domestic ruminants. *Cooperia* spp. show worldwide distribution but abundance of individual species is influenced by climate conditions, in particular rainfall and temperature. In Europe and Australia, *Cooperia oncophora* is most abundant while in tropical and subtropical regions the species *Cooperia pectinata* and *Cooperia punctata* are more abundant. *Cooperia spatulata* appears to have a similar distribution as the latter but is considerably less frequently reported. The rare reports of *C*. *spatulata* were explained by the fact that they are very similar in morphology to *C*. *punctata* and therefore overlooked and/or misidentified [2].

*Cooperia* spp. are considered to be less pathogenic than other GINs in sheep and cattle. However, *C*. *punctata* infections in cattle can reduce feed intake and live weight gain and influence the phosphorus kinetics, reducing phosphor intake, absorption and retention [3]. Infection with *Cooperia* spp. can lead to losses in milk production, weight gain and generally poorer performance [4]. A high *Cooperia* worm burden can lead to anorexia and diarrhea. Remarkably, *C*. *punctata* and *C*. *pectinata* are described to be more pathogenic than *C*. *oncophora* since they penetrate the epithelial surface of the small intestine causing catarrhal enteritis, hypoproteinemia and submandibular edema. Recently, Stromberg *et al*. [5] evaluated the effect of *C*. *punctata* on beef cattle and observed deleterious effects on dry feed intake (-0.68 kg/day) and weight gain (-0.11 kg/day) over a period of 60 days. Moreover, Zebu cattle, which are more resistant to ectoparasites and also some GINs, have higher sensitivity to *Cooperia* spp. compared to crossbreed animals (zebu × taurine) [6].

The intensity of infestation with GIN can be estimated using fecal egg counts but for the genus identification it is necessary to perform larval cultures and microscopic examination of third larvae (L3). This technique is time consuming, identification of larvae requires considerable parasitological expertise [7] and a species differentiation is not possible. The most precise approach for the morphological identification of GIN at species level is the differentiation of adult worms after necropsy. The distinguishing morphological characteristics of the individual species are the shape and length of the spicula as well as the species-specific features of the dorsal ray, genital cone, cephalic vesicle and the number and distribution of cuticular ridges [8, 9].

In some cases, there is disagreement regarding the validity of individual species. For example, the status of *Cooperia mcmasteri* was discussed repeatedly as already reviewed by Allen and Becklund [10] who themselves reported non-overlapping length of spicula between individuals assigned to this species and *Cooperia surnabada*. However, the authors did not state a clear opinion if they consider this difference sufficient to establish two valid species. They summarized that LeRoux [11] and Ault [12] considered *C*. *mcmasteri* a synonym of *C*. *surnabada* while Travassos and Cruz [13] and Skrjabin *et al*. [14] argued that both represent valid species. Later, Karamendin [15] again stated that they are synonyms. Using mating experiments, Isenstein [16] concluded that *C*. *surnabada* and *C*. *oncophora* are, despite minor differences in their cuticular ridge patterns, most likely polymorphs of the same species. In contrast, Lichtenfels [9] listed *C*. *mcmasteri* as a synonym of *C*. *surnabada* but reported differences in the cuticular ridge patterns between *C*. *surnabada* and *C*. *oncophora* and stated that they are valid species. In two independent molecular approaches, Humbert & Cabaret [17], using random amplified polymorphic DNA (RAPD), and Newton *et al*. [18], using comparison of ITS-2 sequences, showed that differences between *C*. *surnabada* and *C*. *oncophora* are considerably smaller than those between other member of the genus and assumed them to be morphotypes of *C*. *oncophora*.

Such data suggests the presence of different morphotypes within a species and/or occurrence of cryptic species with very similar or even undistinguishable morphology. Accurate genus or species identification is essential not only for epidemiological studies but also due to the differences in the pathogenicity between closely related GINs. Furthermore, this is relevant for the implementation of suitable control strategies since efficacy of anthelmintics and occurrence of anthelmintic resistance varies between species and all this information is required to provide optimized treatment recommendations [19–23].

Ribosomal internal transcribed spacer (ITS-1 and ITS-2) sequences are the most commonly used markers for GIN identification since they represent highly variable nuclear loci flanked by conserved regions where primers with broad species specificity can be placed [24]. In accordance, most reports dealing with molecular identification of *Cooperia* spp. have relied on the ITS-2 locus [25–27].

Molecular tools are able to (i) identify cryptic species using highly variable loci suitable for barcoding and (ii) describe phylogenetic relationships using moderately variable genes leading to (iii) specific information about species identities. Additional molecular data may furthermore lead to the development of improved diagnostic methods to discriminate morphologically similar or identical species and perform diagnosis to the species level using larvae or eggs. Genetic markers in the ITS-1 and ITS-2 or external transcribed spacer (ETS) have been used successfully for nematode identification [28–32]. The use of mitochondrial DNA (mtDNA) as source of species-specific markers has been described as a more sensitive methodology to measure evolutionary changes in short periods of time since evolution of mitochondrial sequences is considered to be faster compared to nuclear sequences [33]. In consequence, mtDNA markers represent a more suitable tool for distinguishing closely related nematode species using barcoding approaches [24].

Regarding the genus *Cooperia*, for most of the morphologically described species no or only scarce genetic information is available. The primary aim of this study was to obtain molecular markers to discriminate *Cooperia* species infecting cattle using either morphologically identified adult specimen or mono-species isolates passaged in experimental infections. In particular, adult specimen of *C*. *punctata*, *C*. *pectinata* and *C*. *spatulata* (all from Brazil) were analyzed in comparison with data from larvae using a *C*. *punctata* isolate from Mexico and three *C*. *oncophora* isolates from the UK and New Zealand. The data were used to evaluate the validity of the *Cooperia* species mentioned above and to reconstruct their evolutionary history.

## Materials and methods

### Morphological identification of *Cooperia punctata*, *Cooperia pectinata* and *Cooperia spatulata*

Ethanol-preserved nematode specimens, previously obtained in a controlled anthelmintic resistance test performed in 2015 in Brazil, were used. All *Cooperia* specimen were collected from a single calf and were phenotypically resistant to doramectin and moxidectin.

For this study, 43 male specimens of *C*. *punctata*, 30 of *C*. *pectinata* and 12 of *C*. *spatulata* were obtained from one single host. The specimens were manually cut in two parts: the posterior region was cleared in phenol alcohol (80% melted phenol and 20% ethanol) for examination of the morphological features of spicules, and the anterior and middle regions were fixed in 70% ethanol for the molecular studies.

The morphology and morphometry of the spicules were the criteria to differentiate the species of *Cooperia*, using a compilation of the ‘Key to described species of *Cooperia*’ [34], the ‘Illustrated key to C*ooperia* spp. of North American ruminants’ [9] and the ‘Key to the African species of the genus *Cooperia*’ [8]. To morphologically distinguish *C*. *punctata* and *C*. *spatulata*, the criteria described by Walker and Becklund [35], comparing the concavity and the ventral flange of the spicules were applied. Since the number of *C*. *spatulata* was much lower than the number of *C*. *punctata* and *C*. *pectinata*, worms were initially sorted roughly according to their size. Then, the largest (presumably *C*. *pectinata* and *C*. *spatulata*) and the smallest worms (presumably *C*. *punctata*) were subjected to clearance in phenol alcohol. All morphometric analysis were performed using a LEICA DMi8 optical microscope and the LAS X Measurements software.

### Laboratory isolates of *Cooperia oncophora* and *Cooperia punctata*

Larvae from one fully drug susceptible *C*. *oncophora* isolate (C.o. sen initially originally obtained from the Central Veterinary Laboratory at Weybridge, UK) and two drug resistant isolates, *C*.*o*. IVMres (ivermectin resistant) [36] and *C*.*o* NZres (highly resistant to ivermectin and benzimidazoles) [37] were used.

Furthermore, larvae from a *C*. *punctata* isolate (*C*. *p*. CEIEGT-FMVZ-UNAM), originally isolated in Mexico by the Centre for Research, Teaching and Extension in Tropical Livestock of the Faculty of Veterinary Medicine of the National Autonomous University of Mexico (CEIEGT-FMVZ-UNAM) were included [38]. The complete resistance status of this isolate is currently not known but it was isolated after treatment of cattle with ivermectin. Data from necropsies indicated that *C*. *p*. CEIEGT-FMVZ-UNAM is a pure *C*. *punctata* isolate using the identification key provided by Gibbons [8].

The laboratory isolates were regularly passaged at the Institute for Parasitology and Tropical Veterinary Medicine, Freie Universität Berlin, Germany. Helminth-free calves were orally infected with 30,000–40,000 L3. After 14–21 days, feces from the infected calves were collected and fecal cultures were performed. After 10 days cultivation at 25 °C and 80% humidity, larvae were recovered and purified using the Baermann funnel system and stored in ventilated cell culture flask at 8–10 °C until further use.

### DNA Extraction

For DNA isolation, the ethanol was removed and the worms were washed with sterile water and transferred individually into reaction tubes. DNA was extracted using the NucleoSpin^®^ *Tissue XS* kit (Macherey-Nagel). DNA from larvae was extracted using the NucleoSpin Tissue Kit (Macherey-Nagel). Both procedures were carried out according to the manufacturer’s protocols. DNA was eluted in 10 μl (Tissue XS kit) or 25 μl (Tissue kit) elution buffer and stored at -20 °C until further use.

### PCRs and cloning

PCRs for nuclear genes were conducted using (i) a combination of the forward and reverse primers from two previously published primer pairs flanking a partial ITS-1/5.8S rRNA/complete ITS-2 region [27, 39] and the isotype-1 β-tubulin gene [40] (S1 Table). For amplification of the mitochondrial small subunit rRNA region (12S rRNA) a new primer pair was designed (S1 Table). These three PCR reactions contained 0.2 mM dNTPs, 250 nM of each primer, 0.4 U Phusion Hot Start II High-Fidelity DNA polymerase (Thermo Scientific) and 2 μl template DNA in 20 μl 1×HF buffer. After an initial denaturation at 98 °C for 30 s, 40 cycles of denaturation at 98 °C for 10 s, annealing at a primer specific temperature for 30 s followed by elongation at 72 °C for 30–45 s were carried out. Primer sequences, annealing temperatures and elongation times are listed in S1 Table.

Additionally, a degenerated primer pair for cytochrome oxidase 2 (*cox-2*) (S1 Table) was designed based on the mitochondrial genomes of *C*. *oncophora* (GenBank accession number AY265417/GQ888713). This PCR was performed using the AccuPrime^™^ *Taq* DNA Polymerase System and the reaction contained 1.2 μM of each primer, 1.6 U AccuPrime^™^ Taq DNA Polymerase in 25 μl 1×AccuPrime^™^ PCR Buffer II with a final MgCl_2_ concentration of 3.9 mM. After initial denaturation at 95 °C for 2 min, 40 cycles were performed with denaturation at 95 °C for 15 s, annealing at 49 °C for 20 s and elongation at 68 °C for 30 s.

PCR products were analyzed using electrophoresis in 1.0–1.5% agarose gels and amplicons were purified from agarose gels using the Zymoclean^™^ Gel DNA Recovery Kit or directly from the PCR reaction using DNA Clean & Concentrator^™^-5 (Zymo Research, Germany). Purified fragments were ligated into the StrataClone Blunt PCR Cloning Vector pSC-B-amp/kan (Agilent) and transformed into StrataClone SoloPack Competent *Escherichia coli* cells according to the manufacturer’s protocol. Plasmid DNA was purified using the EasyPrep^®^ Pro kit (Biozym) and sent for sequencing to LGC Genomics.

### Sequence comparisons and phylogenetic analyses

Sequences from the present study were analyzed together with sequences previously deposited in GenBank. As outgroups, a single sequence per gene from the species *Haemonchus contortus*, *Haemonchus placei*, *Teladorsagia circumcincta*, *Trichostrongylus vitrinus* and *Trichostrongylus axei* were initially included. Accession numbers of all sequences used in the study are available from S2 Table. The ITS and 12S rRNA sequences were aligned using MAFFT (multiple sequence alignment using fast Fourier transformation) in the Q-INS-I modus that takes predicted RNA secondary structures into account [41]. For the 12S rRNA, it was chosen to align gappy regions anyway while gappy regions were left for ITS regions. The *cox-2* and isotype 1 β-tubulin gene sequences were aligned using the M-COFFE modus of T-Coffee (Tree-based Consistency Objective Function for alignment Evaluation) [42] and manually edited to ensure that codons were not interrupted by gaps. For calculation of relative identity (%) between sequences, alignments were analyzed using the dist.dna function in the ape 4.0 (Analyses of Phylogenetics and Evolution) package [43] in R 3.3.1 statistics software. Identities were calculated as “raw” identities and pairwise deletion of positions with gaps was turned off. Comparisons of sequences were sorted into the intra-species groups *C*. *oncophora*, *C*. *pectinata*, *C*. *punctata* and *C*. *spatulata*. In addition, all possible comparisons between these groups were used as additional categories. The identity in percent for all these comparisons within the genus *Cooperia* were compared using One-way ANOVA followed by a Bonferroni post-hoc test in GraphPad Prism 5.03 comparing all possible combinations of groups. All p-values below 0.05 were considered to be statistically significant.

Phylogenetic analyses were initially conducted on a single gene level. First, substitution saturation tests were conducted according to Xia et al. [44] using DAMBE 5 (Data Analysis in Molecular Biology and Evolution) software [45]. Phylogenetic trees were calculated in RAxML 8.2.9 (Randomized Axelerated Maximum Likelihood) [46] on the CIPRES (Cyberinfrastructure for Phylogenetic Research) Science gateway server [47]. For *cox-2*, two separate partitions for codon positions 1 and 2 and codon position 3 were used. For a multi-locus analysis, partitions for (i) ITS-1 and 2, (ii) isotype 1 β-tubulin, (iii) 12S rRNA, (iv) *cox-2* codon positions 1 and 2 and (v) cox-2 codon position 3 were analyzed. For each partition, GTRGAMMA models with 25 substitution rate categories were fitted. Initially, a best maximum-likelihood tree was estimated with a rapid bootstrapping analysis (-f a option in RAxML). The software was set to use 1000 rapid bootstraps without stopping criterion. In a second step, the tree obtained in the bootstrapping analysis was used as additional input to constrain the tree topology. An independent branch-support for all nodes was calculated using the Shimodaira-Hasegawa modification of the likelihood-ratio test (-f J option in RAxML). Phylogenetic trees were visualized in Mega 7 (Molecular Evolutionary Genetics Analysis) [48] and further edited in CorelDraw XV.

### Ethical statement

All animal experiments conducted in Germany were in agreement with the regulation on animal protection of the European Union (directive 2010/63/EU) and Germany (“Tierschutzgesetz”) and were approved by the Landesamt für Gesundheit und Soziales (LAGeSo) Berlin under the reference number L/8810.

Experiments performed in Brazil followed the Brazilian Law No. 11,794, dated October 8, 2008 (Lei Arouca), regulated by Decree No. 6899, of July 15, 2009. The protocol was approved by the Ethics Committee on the Use of Animals (CEUA) of the UFMS (protocol 683/2015) and the experimental stage was initiated only after approval of the protocol.

## Results

### Morphological identification

**Fig 1:**
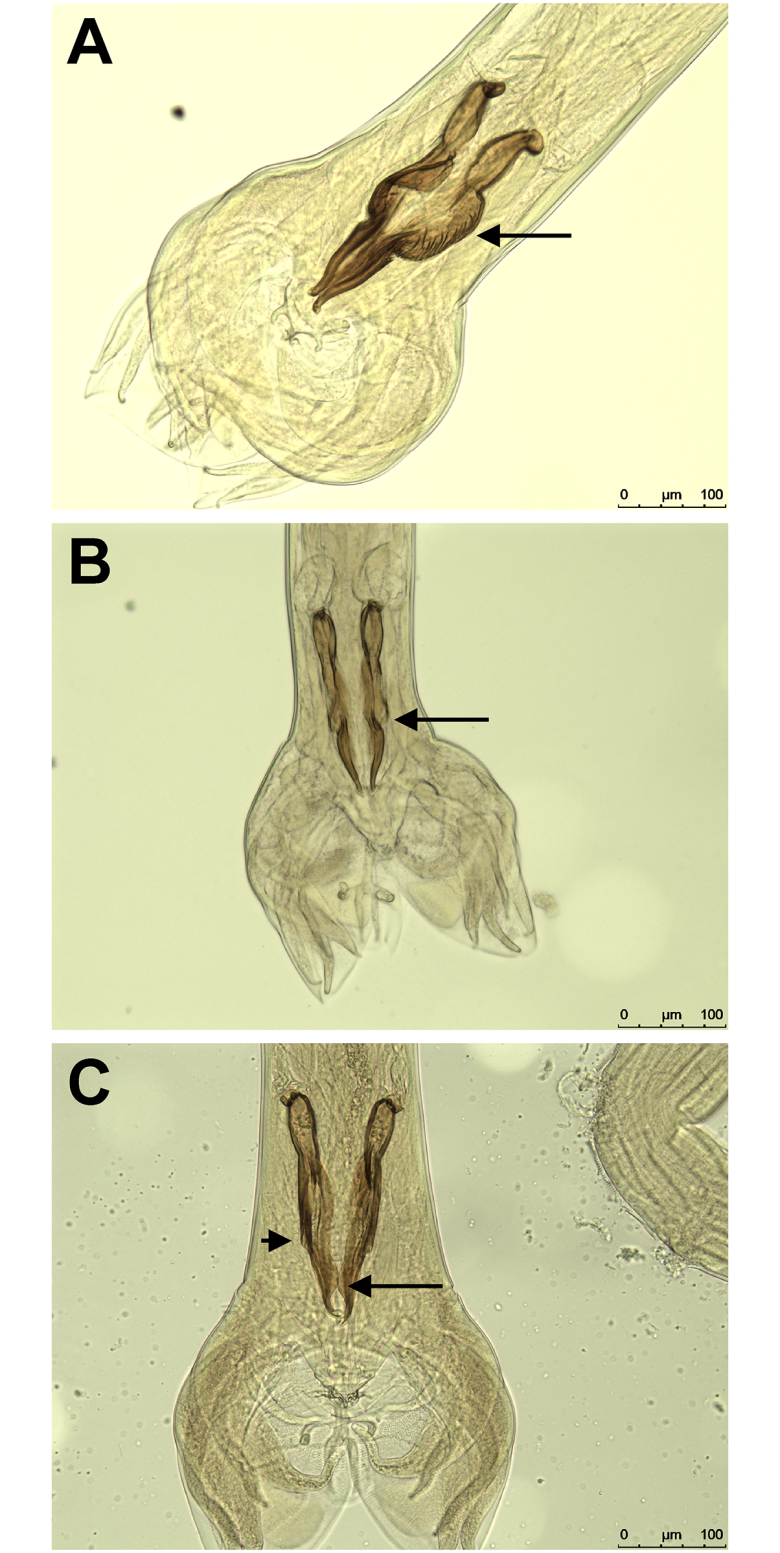
Ventral view of spicules of *Cooperia* spp. from a Brazilian calf. (A) *Cooperia pectinata* males have large spicules with corrugated edges in the middle third (arrow). (B) *Cooperia punctata* spicules show a large concavity near the middle, which has a distinct border and a lateral narrow projection. (C) *Cooperia spatulata* spicules have a small concavity (small arrow) and a large ventral flange (large arrow).

**Fig 2:**
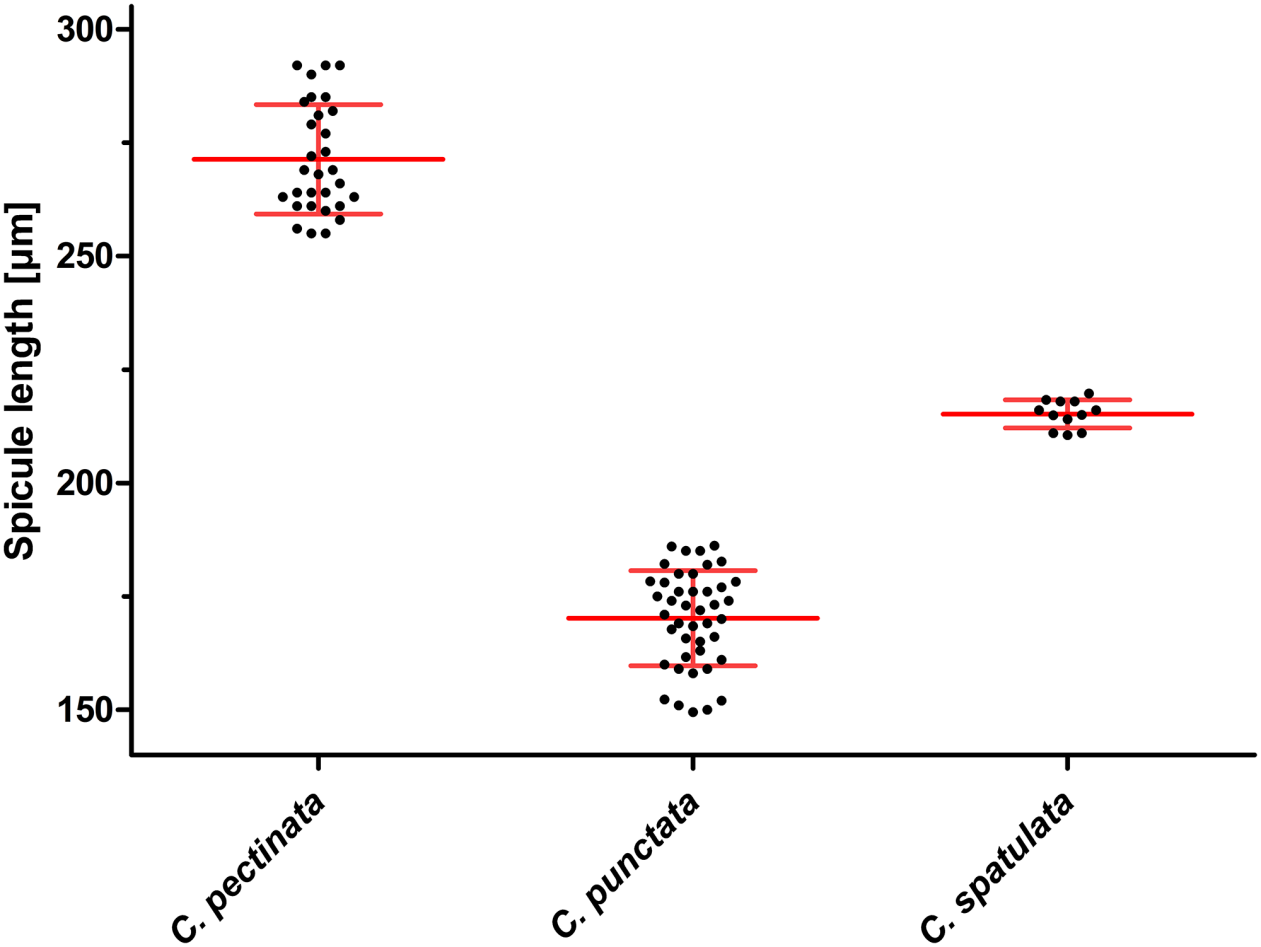
Scatter plot of spicule length of *Cooperia pectinata*, *Cooperia punctata* and *Cooperia spatulata* collected from a calf in Brazil. Mean spicule length of 30 *C*. *pectinata*, 43 *C*. *punctata* and 12 *C*. *spatulata* was plotted and compared using One-Way-ANOVA and Bonferroni post-hoc tests. All groups were significantly different with p<0.0001 but it must be kept in mind that the *C*. *punctata* worms were preselected according to a smaller over-all length which clearly introduces a systematical bias. Individual values are shown in black while mean ± SD are indicated in red.

The morphologic identification (Fig 1) and morphometric comparison (Fig 2) of the spicula of *C*. *pectinata*, *C*. *punctata* and *C*. *spatulat*a collected from a single Brazilian calve revealed clear differences that allowed separation of the worms into three categories (Fig 1). This is also reflected by significant morphometric differences but it must be kept in mind that worms had been preselected according to size in order to find sufficient numbers of *C*. *spatulata*, which most likely introduced a systematic bias. However, it must also be stated that no *C*. *spatulata* were found in the group of small worms and no *C*. *punctata* in the group of large worms. Thus, there was a clear morphological distinction between *C*. *punctata* and *C*. *spatulata* although the morphometric data most likely overestimate this difference.

### PCR results

For adult worms collected in Brazil and morphologically identified as *C*. *punctata*, *C*. *pectinata* or *C*. *spatulata*, PCR products for the nuclear markers partial ITS-1/5.8S rRNA/complete ITS-2 (815–822 bp excluding the primers) and partial isotype 1 β-tubulin gene (385–400 bp) as well as the mitochondrial 12S rRNA (170 bp) and *cox-2* (406 bp) genes were amplified, cloned and sequenced for at least five individuals per species. For at least four individual worms per species, all four amplicons were sequenced. In addition, sequences for two larval pools of the isolates C.o sen and C.o IVMres, as well as one larval pool of C.o NZres and *C*. *punctata* CEIEGT-FMVZ-UNAM were obtained. All sequences were deposited in GenBank under the accession numbers provided in S2 Table.

### Genetic distance of nuclear and mitochondrial marker sequences

**Fig 3:**
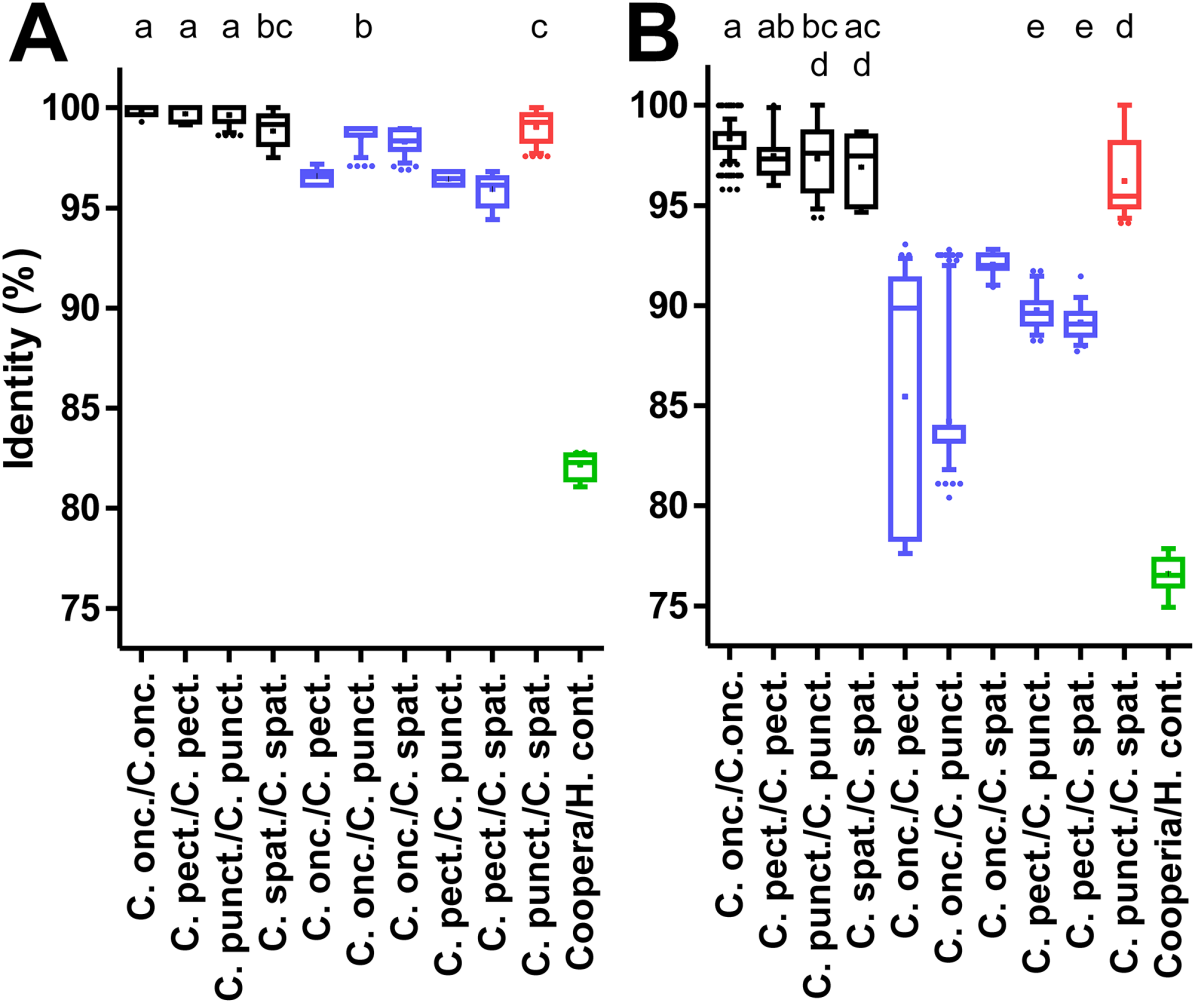
Pairwise sequence identities for intra- and inter-species comparisons for nuclear ITS-2 and isotype 1 β-tubulin loci. Identities were calculated from multiple sequence alignments for the ITS-2 region (A) and a partial genomic isotype 1 β-tubulin gene containing two introns (B). Data are presented as box plots showing the median and 25 and 75% percentiles. Whiskers represent 5–95% quantiles and outliers are represented by dots while the cross indicates the mean. Intra-species comparisons are shown in black, inter-species comparisons within the genus *Cooperia* in blue, comparisons between *C*. *punctata* and *C*. *spatulata* in red. All comparisons between a *Cooperia* sequence and a representative *H*. *contortus* sequence are indicated in green. Accession numbers for all sequences that were included in the analysis are available from S2 Table. Datasets that do not share at least one of the index letters (a-c in panel A and a-e in panel B) are significantly different (p<0.05) from each other in a Kruskal-Wallis test followed by a Dunn’s post hoc test between all *Cooperia* groups.

**Fig 4:**
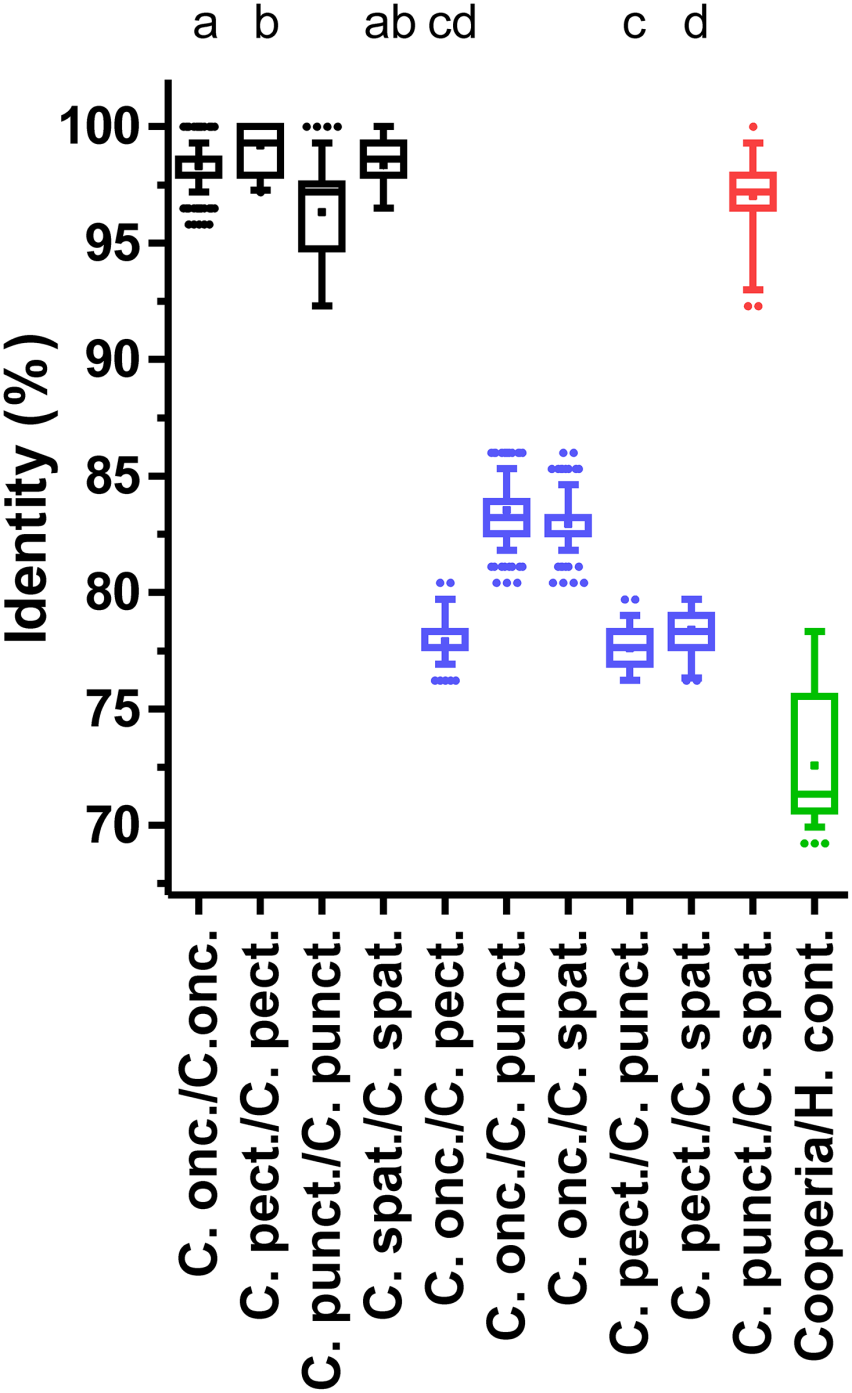
Pairwise sequence identities for intra- and inter-species comparisons at the 12S mitochondrial locus. Identities were calculated from a multiple sequence alignment of the partial mitochondrial 12S rRNA sequence. Identities are shown as box plots with whiskers representing 5–95% quantiles. Dots indicate outliers and the cross marks the means of the datasets. Intra-species comparisons are colored in black, inter-species comparisons within the genus *Cooperia* in blue and comparisons between *C*. *punctata* and *C*. *spatulata* in red. Comparisons between any *Cooperia* sequence and a representative *H*. *contortus* sequence are indicated in green. All accession numbers for included sequences are provided in S2 Table. Datasets without at least one of the index letters (a-d) in common are significantly different (p<0.05) from each other as revealed by Kruskal-Wallis test followed by a Dunn’s post hoc test between all *Cooperia* groups.

**Fig 5:**
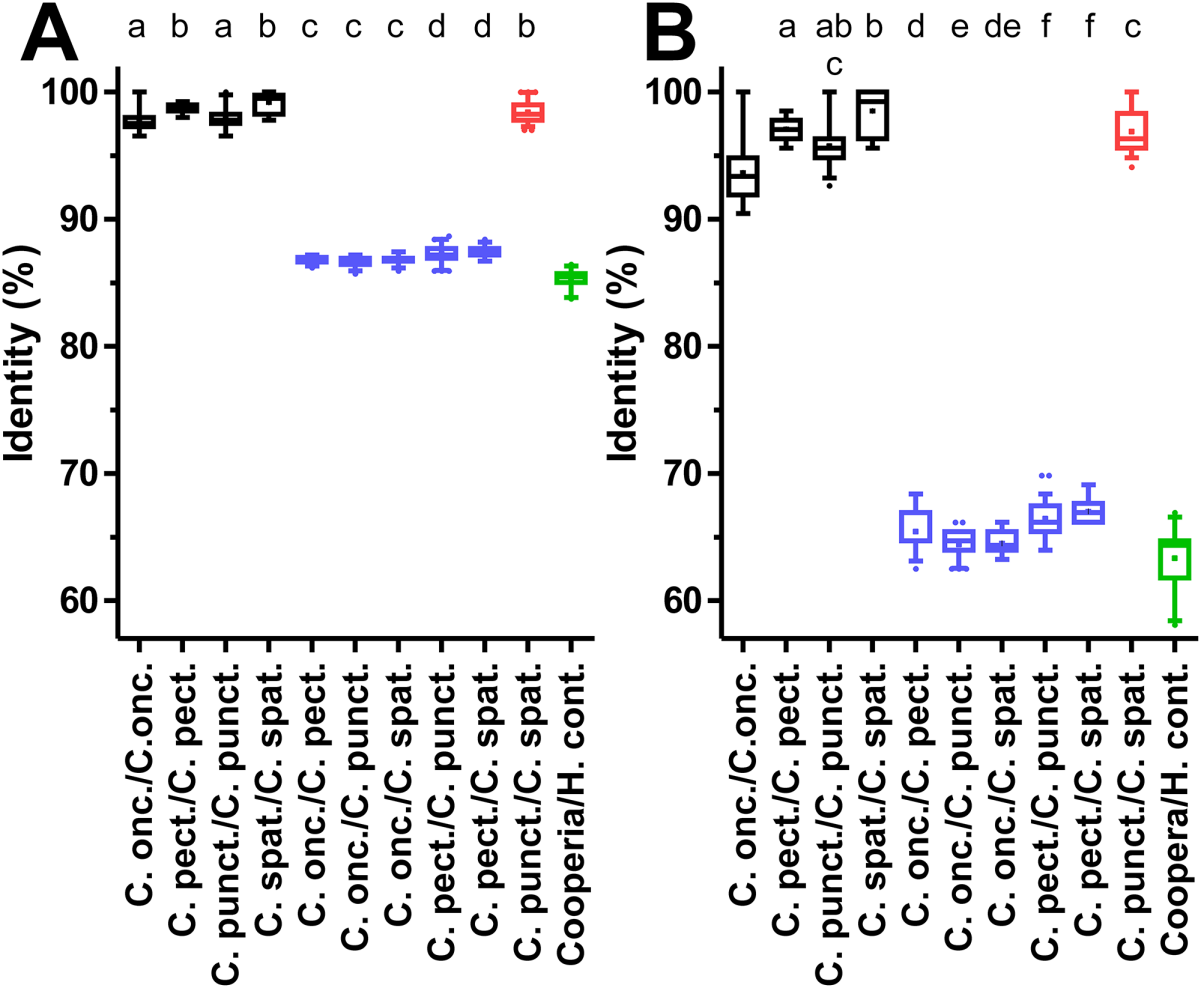
Pairwise sequence identities for intra- and inter-species comparisons at the mitochondrial *cox-2* locus. Pairwise identities are plotted as box plots for a partial cytochrome oxidase 2 (*cox-2*) sequence (A) and the third codon position of the same fragment (B). Whiskers indicate 5–95% quantiles with outliers represented by dots. The mean of each dataset is marked by a cross. Intra-species comparisons and inter-species comparisons within the genus *Cooperia* are shown in black and blue, respectively. Comparisons between *C*. *punctata* and *C*. *spatulata* are drawn in red. All comparisons between a *Cooperia* sequence and a representative *H*. *contortus* sequence are plotted in green. All accession numbers for sequences used in the analysis are given in S2 Table. Index letters (a-d in panel A and a-f in panel B) are used to indicate significant differences between groups as revealed by Kruskal-Wallis test followed by a Dunn’s post hoc test between all *Cooperia* groups. Only groups sharing no index letter are significantly different (p<0.05).

Initially, the ITS-2, isotype 1 β-tubulin gene, 12S mitochondrial rRNA and *cox-2* loci were analyzed to identify those genes that have the potential to discriminate between species and genera. For this purpose, sequences were individually aligned for each locus with available GenBank entries from the same species and together with a single sequence from *H*. *contortus*, *H*. *placei*, *T*. *circumcincta*, *T*. *vitrinus* and *T*. *axei*. From the alignments, the pairwise identity in percent was calculated and plotted for the different intra- and inter-species comparisons (Figs 3–5). In addition, the comparison with a single representative sequence from *H*. *contortus* was included in the graphs. For the ITS-2 sequences, intra-species comparisons (97.5–100% identity) and inter-species comparisons within the genus *Cooperia* (96.2–100% identity) were clearly overlapping (Fig 3A). In particular, comparisons between *C*. *punctata* and *C*. *spatulata* revealed the highest degree of identity between 97.6 and 100%. In contrast, there was a markedly higher difference to the *H*. *contortus* sequence (81.1–82.8% identity), which is much lower than the intra-genus comparisons. Results for other species outside of the genus *Cooperia* were very similar.

For the second nuclear marker, the partial isotype 1 β-tubulin gene sequence, variation in identities observed for the different comparisons appeared to be considerably larger than for the ITS-2 region (Fig 3B). In contrast to the ITS-2 region, the intra-species comparisons (94.7–100% identity) and the inter-species comparisons (77.6–93.1% identity) were not overlapping but the distance between both ranges was only small. However, the comparison between *C*. *punctata* and *C*. *spatulata* revealed much higher identity ranging between 94.1% and 100% than comparisons between other *Cooperia* sequences. This was not significantly different from the intra-species comparisons for *C*. *punctata* (94.4–100%) and *C*. *spatulata* (94.7–100%) (Fig 3B). Comparison between *Cooperia spp*. and the *H*. *contortus* sequences showed 74.9–77.9% identity, which slightly overlaps the range observed for comparisons within the genus *Cooperia* (77.6–100%).

The mitochondrial 12S rRNA gene comparisons (Fig 4) revealed identities between 92.3 and 100% for intra-species comparisons. Inter-species comparisons within the genus *Cooperia* showed between 76.2% and 100% identity. Again, the highest inter-species identities were observed for the comparisons between *C*. *punctata* and *C*. *spatulata* with a range of 92.3–100%. If this comparison was excluded, inter-species identities in the genus *Cooperia* were between 76.2 and 86.0%. With exception of the *C*. *punctata*/*C*. *spatulata* species pair, a clear distinction of species was possible using the 12S rRNA gene sequences. Comparisons of *Cooperia* sequences to the *H*. *contortus* sequence showed identities in the range of 69.3–78.3%. The variability in identities were higher than observed for the ITS-2 sequences but much smaller than for the isotype 1 β-tubulin gene (Fig 4).

Finally, the *cox-2* gene analysis was conducted independently for the complete sequence (Fig 5A) and only for the third codon position (Fig 5B). Comparison of Fig 5A and 5B directly shows that most of the variability between sequences can be attributed to differences in the third codon position. In both analyses, there was a clear difference between intra-species and inter-species comparisons within the genus Cooperia—again except of the comparison between *C*. *punctata* and *C*. *spatulata*. For all three codon positions, intra-species comparisons were in the range 96.6–100% while inter-species comparisons revealed only 85.7–88.7% identity with exception of the comparison between *C*. *punctata* and *C*. *spatulata* which showed 97.0–100% identity which is in the same range as observed for the intra-species comparisons (Fig 5A). Analyses using only the third codon position revealed very similar results but variabilities and differences between intra- and inter-species were in general higher (Fig 5B).

For both mitochondrial sequences that were analyzed in this study, there was no clear distinction between inter-species comparisons within the genus *Cooperia* and comparisons between *Cooperia* spp. and *H*. *contortus* (Figs 4 and 5).

### Phylogenetic analysis

Using the same alignments that were analyzed to calculate relative identities, phylogenetic trees were calculated using a single gene per tree. Only for the ITS region, not only the ITS-2 but the partial ITS-1, 5.8S rRNA and ITS-2 were aligned and used to calculate the tree shown in S1 Fig while S2–S4 Figs show the trees for the partial isotype 1 β-tubulin gene, the mitochondrial 12S rRNA and *cox-2* genes, respectively. In the ITS analysis (S1 Fig), the genus *Cooperia* forms a well-defined operational taxonomic unit (OTU) with high support by rapid bootstrapping and the Shimodaira-Hasegawa test. Support for the species *C*. *pectinata* is also very high (≥95% in both tests) while *C*. *oncophora* support is considerably lower (89 and 91% with the bootstrapping and Shimodaira-Hasegawa test, respectively). The third cluster in the genus *Cooperia* contains all the sequences from the two morphospecies *C*. *punctata* and *C*. *spatulata*. Support for is cluster is moderate, 82% in the rapid bootstrapping analysis and 97% using the Shimodaira-Hasegawa test. Neither the morphotypes nor the geographical origin (Brazil vs. Mexico) formed subclusters. In contrast, they appeared to be randomly distributed within the *C*. *punctata*/*C*. *spatulata* cluster (S1 Fig).

In the analysis using the isotype 1 β-tubulin gene (S2 Fig), only *H*. *contortus* was included as outgroup since the intron positions were not identical for the different species which led to unreliable alignments. This outgroup connected to the *Cooperia* sequences within the *C*. *punctata*/*C*. *spatulata* cluster splitting this cluster into two parts. Within the *C*. *punctata*/*C*. *spatulata* cluster, again both morphotypes and both countries of origin were scattered all over the cluster. In contrast, *C*. *oncophora* and *C*. *pectinata* formed clearly defined and well supported clusters, again with higher statistical support for *C*. *pectinata* than for *C*. *oncophora*.

In the analysis of the mitochondrial 12S rRNA sequences, a large number of *C*. *oncophora* sequences that were available in GenBank was included (S3 Fig). In this analysis, *C*. *pectinata* grouped together with the *Haemonchus* sequences and not with the other *Cooperia* sequences. The *C*. *pectinata* OTU was highly supported and the large *C*. *oncophora* OTU also showed good but again lower statistical support. In contrast, all sequences from the morphospecies *C*. *punctata*/*C*. *spatulata* again formed a single cluster. However, support for this cluster is quite poor (27% in rapid bootstrapping analysis and 81% in the Shimodaira-Hasegawa test). Again, there was no subcluster showing geographical or morphotype-specific patterns.

The *cox-2* analysis (S4 Fig) revealed three very homogenous clusters of *Cooperia* species, again representing *C*. *pectinata*, *C*. *oncophora* and *C*. *punctata*/*C*. *spatulata*. However, two of the outgroup sequences were placed within the *C*. *pectinata* cluster but this combined cluster showed only low support. In contrast, the *C*. *oncophora* and *C*. *punctata*/*C*. *spatulata* clusters were very highly supported in both statistical approaches (98–100%). The morphotypes *C*. *punctata* and *C*. *spatulata* as well as the geographic origins appeared to be distributed all over the corresponding cluster.

**Fig 6:**
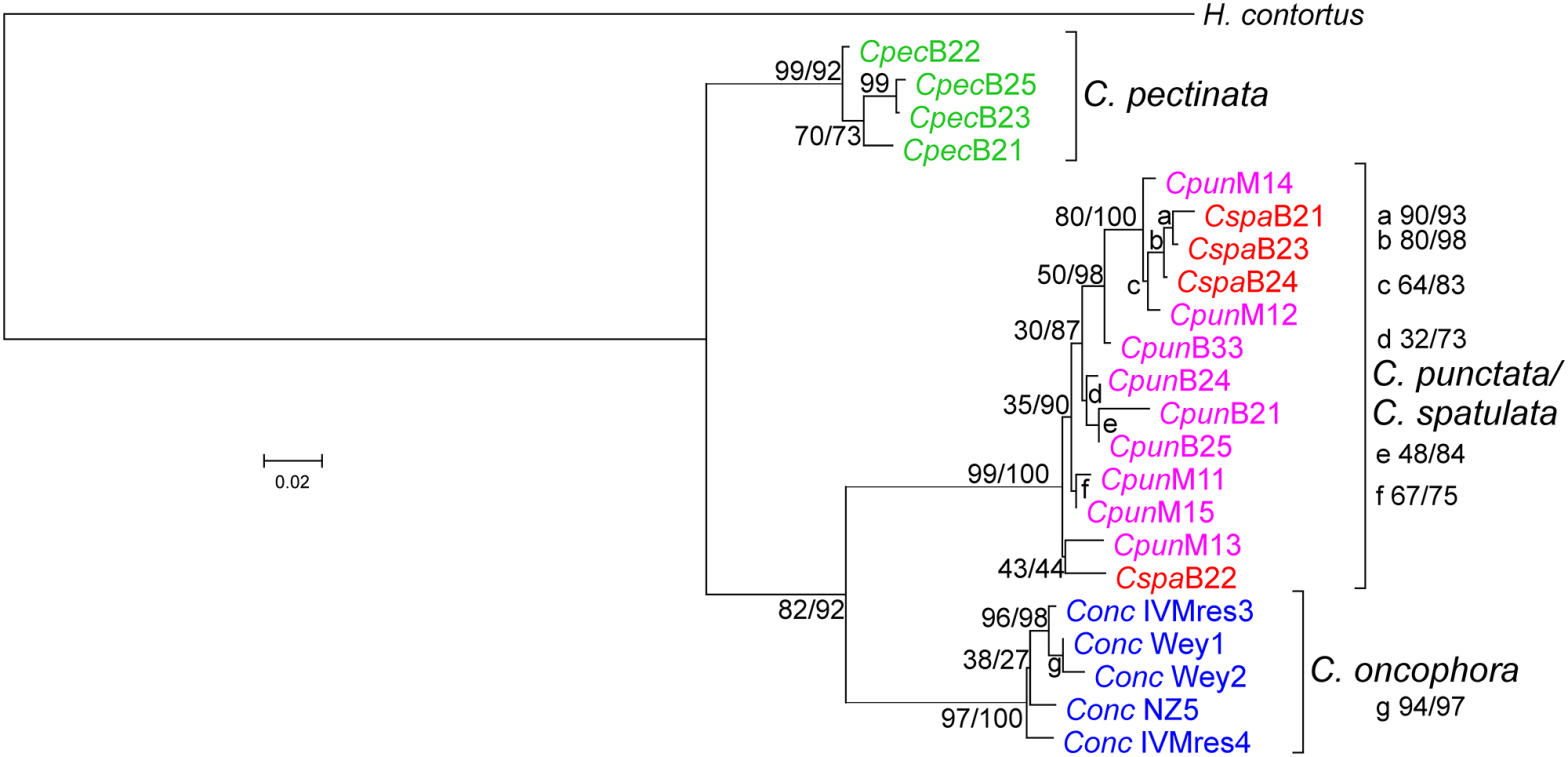
Multi-locus phylogenetic analysis of *Cooperia* species infecting cattle. Sequences were aligned using M-Coffee (partial isotype 1 β-tubulin and mitochondrial *cox-2* genes) or MAFFT (partial ITS-1, complete 5.8S, ITS-2 fragment and partial mitochondrial 12S rRNA genes). Protein coding regions were manually edited to ensure that codons were not disrupted by gaps. A phylogenetic tree was calculated using RAxML with one partition per gene except for the *cox-2* gene for which separate partitions for codon positions 1 and 2 and codon position 3 were included. Sequences from *Haemonchus contortus* (accession numbers DQ469245 + KT428386 + EU346694were used as outgroup. Samples were obtained from individual worms identified as *Cooperia pectinata* (*Cpec*, green), *Cooperia punctata* (*Cpun*, magenta) from Brazil, and *Cooperia spatulata* (*Cspa*, red) and from pools of larvae from *Cooperia oncophora* (*Conc*, blue) and *C*. *punctata* from Mexico. Sequences derived from Brazil (B) and Mexico (M) are indicated together with numbers indicating the particular voucher (in combination with *Cooperia* morphospecies and geographical origin. The *C*. *oncophora* and the Mexican *C*. *punctata* samples were obtained from different pools of larvae using isolates that have been characterized as single species isolates. Node support values represent results of the rapid bootstrapping analysis before and of the Shimodaira-Hasegawa likelihood ratio test behind the slash. Accession numbers for all new sequences are available from S2 Table.

Finally, a multi-locus phylogenetic analysis was conducted using only *H*. *contortus* as outgroup due to the difficulties in alignment of the isotype 1 β-tubulin introns. The analysis furthermore included only the sequences from those specimen or larval pools for which sequences for all four loci were available. The resulting tree (Fig 6) showed again excellent support for the three *Cooperia* clusters already observed in the single gene analyses. *Cooperia pectinata* was located at a more basal position and *C*. *oncophora* in a sister position to the cluster containing all *C*. *punctata* and *C*. *spatulata* sequences. As already observed for the individual sequences, the *C*. *spatulata* or *C*. *punctata* sequences did not cluster with each other and the same also holds true for samples from Brazil or Mexico.

## Discussion

The Interim Register of Marine and Nonmarine Genera [49] currently lists 34 accepted species and seven synonyms in the genus *Cooperia* [50]. For most of these species, only a morphological description is available while molecular data are missing or scarce even for several species infecting livestock. To unequivocally identify individual species and their evolutionary history, a combination of sequence analyses using highly and moderately variable DNA loci for barcoding and calculation of phylogenetic relationships is required, respectively.

The morphology and morphometry of the spicules of males have been previously described in the literature as criteria allowing the differentiation between *C*. *pectinata*, *C*. *punctata* and *C*. *spatulata* [2, 8, 9, 34]. Here, the morphology of the spicules was used as the primary criterion to assign specimen to a particular species. Subsequent morphometric analysis revealed non-overlapping ranges of spicule lengths and significant differences between groups. This is in contrast to previous reports on the length of the spicules that suggested overlapping ranges for *C*. *punctata* and *C*. *spatulata* while spicules of *C*. *pectinata* were larger than those of the other species in all studies. Comparisons of average lengths and ranges observed in this study and described in the literature are presented in Table 1. However, it must be clearly stated that the ranges of spicule length varied widely between different studies. The small ranges and clear separation observed in the present study may have been influenced by sampling bias, since the parasites used in this study were preselected according to their overall length in order to collect enough specimens of the rare *C*. *spatulata* for subsequent molecular analysis. Another bias was presumably introduced by the fact that all specimens of *Cooperia* included in this analysis were collected from the same individual host. This limitation was simply due to the fact that *C*. *spatulata* was found only in very few animals and only with low intensity. In order to collect and morphometrically analyze enough *C*. *spatulata* in a feasable time, the calf with the highest frequency of *C*. *spatulata* in the *Cooperia* population was chosen. Therefore, only a single population of *C*. *spatulata* was included and not a broad representation of different populations, which might show considerably higher variability. A related issue is the fact that only male worms were included in the present study. Although faint differences in morphology of female *C*. *spatulata* and *C*. *punctata* have been described, these differences all rely on the longitudinal ridge patterns of the cuticle. In order to determine these differences, it would have been necessary to prepare cross sections (e.g. after embedding in paraffin), screen hundreds of cross sections to identify female *C*. *spatulata* and then isolate DNA from the (embedded) material. This was not possible within the frame of the present project.

**Table 1:** Average, minimum and maximum spicule length of *Cooperia pectinata*, *Cooperia punctata* and *Cooperia spatulata* reported in different studies.

Study	Spicule length (μm)			
	Average	Minimum	Maximum	n
*Cooperia pectinata*				
This study	271.9	252.4	294.5	30
Lichtenfels [9]	n.a.^a^	220	390	n.a.
Schwartz [34]	n.a.	240	300	n.a.
*Cooperia punctata*				
This study	170.3	149.5	186.1	43
Lichtenfels [9]	n.a.	125	214	n.a.
Schnyder [51]—original description	n.a.	136.0	149.0	n.a.
Schwartz [34]	n.a.	120.0	150.0	n.a.
Walker and Becklund [35]		167.0	214.0	33
*Cooperia spatulata*				
This study	215.9	210.5	219.7	12
Arantes *et al*. [52]	215.0	185.0	261.0	n.a.
Baylis [53]—original description	n.a.	230.0	290.0	n.a.
Paloschi and Honer [54]	217.2	185.0	260.0	57
Walker and Becklund [35]	n.a.	184.0	279.0	39

^a^Not available.

For molecular analysis of the different specimens, cloned PCR products were used and only a single clone per individual worm was sequenced. Using cloned PCR products offers the advantage of higher quality of sequencing chromatograms and thus lower risks of sequencing errors. However, this is advantage comes at the price that PCR errors might be present in the cloned fragments and thus PCR introduced mutations are considered to be biological variation. There are two considerations that show that PCR induced mutations do not play a relevant role regarding the observed variability. First, the intra- and inter-species variation in the largest amplicon (ITS-1/2 sequence) are very low but nevertheless a slightly higher intra- than inter-species variability was observed. Second, the Phusion High-Fidelity DNA polymerase used herein has an error rate of only 4.4×10^−7^. For the 22 concatenated sequences of approximately 2,165 bp used to calculate the phylogenetic tree in Fig 6, it can therefore be expected that after 40 PCR cycles 3.81% contain one sequencing error, which corresponds to 0.8 sequences. For the whole dataset of approximately 60,550 bp, the probability to have one error is 106.6% and it can therefore be assumed that there might be on average slightly more than one error in a dataset generated using the described methods. Comparing this value with the overall size of the dataset, this error rate obviously does not have any relevant impact on the outcome of the data analyses.

In contrast to the morphological data, no molecular distinction between *C*. *punctata* and *C*. *spatulata* was possible with any of the four marker sequences analyzed. This was observed using both, the calculation of raw percent identity as well as maximum-likelihood phylogenetic analyses.

The ITS-1 and ITS-2 sequences showed only minimal differences between the *Cooperia* species suggesting that they are more suitable for genus than for species identification. This observation is presumably typical for many trichostrongyloid nematodes. For instance, Chaudhry *et al*. [55] compared ITS-2 sequences from three and four isolates of *H*. *contortus* and *H*. *placei*, respectively. These isolates had various geographical origins. They identified only three single nucleotide polymorphisms (SNPs) that they considered to be fixed between the two species while at 21 positions SNPs occurred within *H*. *contortus* and at 11 positions SNPs were found in *H*. *placei*. The authors then established a pyrosequencing assay to quantify the species in mixed samples using one of the fixed SNPs. However, sequences deposited in GenBank since the manuscript was published reveal that the SNP is not fixed and the nucleotide assumed to be specific for *H*. *contortus* can also occur in *H*. *placei*. This shows that a molecular diagnostic assay relying on such a small number of differences is always risky—particularly if information from reference sequences in GenBank is limited in terms of number and/or origin from different populations. The data shown here also affect the interpretation of recently published nemabiome data, a method that uses deep-amplicon sequencing using next-generation sequencing based on ITS-2 PCRs [56]. In a recent field study, the method was applied to study parasitic nematode diversity in cattle from Canada, the USA and Brazil [57]. The authors have deposited *C*. *spatulata* (accession-no KY741872- KY741875) and C. *punctata* (KY741869- KY741871 and KY741880) ITS-2 sequences in GenBank and these sequence have been included in the pairwise comparison of identities presented in Fig 2 where they did not behave in any way different from the sequences generated in the present study. It would be interesting to compare also the mitochondrial sequences presented here with the type material used in the nemabiome study as well as with additional, independently morphotyped material.

In contrast to ITS-2, clear separation of the species *C*. *punctata*, *C*. *pectinata* and *C*. *oncophora* was possible using two mitochondrial marker sequences and also the nuclear isotype 1 β-tubulin gene. Most variability in the latter stems from the intron sequences. However, differences were considerably more distinct for the mitochondrial marker sequences and in particular the *cox-2* sequences showed very high intra-species homogeneity (>96% identity) while identity between species was never higher than 89%. This difference becomes even more pronounced when looking only at the third codon position for which intra-species comparisons revealed more than 90% identity whereas inter-species values were always below 67%. In contrast to improved ability to distinguish species within a genus, the mitochondrial sequences were not suitable for genus identification. Comparisons between different species within the genus *Cooperia* showed similar degrees of identity as comparisons between *Cooperia* spp. and *Haemonchus contortus*, which even belongs to another family, i.e. Cooperiidae vs. Haemonchidae [1]. In agreement with this observation, the genus *Cooperia* did not form a monophyletic cluster in the phylogenetic analyses of the mitochondrial 12S rRNA and *cox-2* sequences.

The better suitability of mitochondrial markers in comparison to nuclear markers in particular for identification of cryptic species has already been emphasized by Blouin [24]. However, due to the absence of recombination and only maternal heritage, mitochondrial marker sequences alone can give misleading results due to lineage sorting. For instance, in the filarial genus *Onchocerca*, three closely related species have been postulated using mitochondrial markers. *Onchocerca ochengi* and *Onchocera* sp. Siisa infect cattle while *Onchocerca volvulus* is a human parasite. Using mitochondrial sequences, *Onchocerca* sp. Siisa appeared to be at least as closely related to *O*. *volvulus* as to *O*. *ochengi* [58, 59]. In contrast, combined analysis of two mitochondrial and six nuclear markers revealed free interbreeding between *O*. *ochengi* and *Onchocerca* sp. Siisa indicating that the latter is only a specific mitochondrial genotype of *O*. *ochengi* [59]. Only using complete mitochondrial genome sequences, closer relatedness of *O*. *ochengi* and *Onchocerca* sp. Siisa than relatedness to *O*. *volvulus* could be demonstrated and inclusion of partial nuclear genomes allowed the conclusion that the Siisa genotype is in fact only a genotype of *O*. *ochengi* and not a separate species [60]. This example shows the importance to include also nuclear markers before finally deciding about the status of cryptic species.

The data presented here also show that a combined analysis of mitochondrial and nuclear markers is also highly informative regarding the taxonomic status of closely related morphotypes. The proposed species *C*. *punctata* and *C*. *spatulata* show faint but clearly distinct morphological patterns of males (longitudinal ridge pattern and spicula) and females (only longitudinal ridge pattern). The absence of significant differences in sequence identity regarding comparisons of any of the mitochondrial or nuclear marker sequences between *C*. *punctata* and *C*. *spatulata* and very similar variability observed in intra-species comparisons for the other three *Cooperia* spp. is a strong indication that *C*. *punctata* and *C*. *spatulata* represent only morphotypes of the same species. This is also supported by all single and the multi-locus phylogenetic analyses in which sequences from both morphotypes are located in the same cluster and do not form any specific sub-groups within the cluster. The same is also true for *C*. *punctata* sequences obtained from specimen from Brazil and Mexico, which also did not form separate clusters.

Another important argument that *C*. *spatulata* is not a valid species but only a morphotype of *C*. *punctata* is the similarity to *C*. *punctata*, including the overlap of the ranges of spicule lengths [35] and the impossibility to distinguish the species by the genital cones [61]. Previously, different male morphotypes have been described for many trichostrongyloid members of the subfamily Ostertaginae. Dróżdż [62] analyzed 23 species of Ostertaginae and 19 of these had more than one male morphotype. Predominant (major) and rare (minor) morphotypes have frequently even been assigned even to different genera. Using exclusively morphological data, Dróżdż [62] convincingly showed that predominant and rare morphotypes always occur as pairs. The dimorphic species were from the predominant genera *Ostertagia* (rare morph *Skrjabinagia*), *Orloffia* (*Buriatica*), *Teladorsagia* ("Trifurcata" morph), *Marshallagia* (*Grosspiculagia*) and *Spiculopteragia* (*Rinadia*). In contrast, the genera *Camelostrongulus*, *Spiculopteragia*, Mazamastrongylus, *Sarwaria* and *Longistrongylus* apparently lacked such dimorphic genotypes.

## Conclusions

The data presented here shows that the ITS region alone is not sufficient for valid identification of closely related species in a genus of Trichostrongyloidea and suggests that additional markers with better barcoding properties should be used in addition. The molecular comparisons between morphotypes clearly question the validity of the species *C*. *spatulata* and suggest that it is a synonym of *C*. *punctata*. Unfortunately, it is not possible to use type specimen from the original descriptions of the nematode species to perform molecular analyses without destroying such invaluable material. Additional molecular data of specimen that were independently identified as *C*. *spatulata* by several experts in the morphology of Cooperinae and coming from different geographic areas would be an obvious way to solve this and similar taxonomic issues.

## Supporting information

S1 Table Primers and PCR conditions. (PDF)

S2 Table GenBank accession numbers for all sequences included in the phylogenetic analyses and not provided the figures. (PDF)

S1 Fig Phylogenetic analysis using partial ITS-1, complete 5.8S rRNA and ITS-2 sequences. Sequences were aligned using MAFFT and a phylogenetic tree was calculated using RAxML without partitioning the data. Sequences from *Teladorsagia circumcincta*, *Trichostrongylus vitrinus*, *Trichostrongylus axei*, *Haemonchus contortus* and *Haemonchus placei* were included as outgroups. Samples were obtained from individual worms identified as *Cooperia pectinata* (*Cpec*, green), *Cooperia punctata* (*Cpun*, magenta), *Cooperia spatulata* (*Cspa*, red) and *Cooperia oncophora* (*Conc*, blue). Sequences derived from Brazil (B) and Mexcico (M) are indicated together with numbers indicating the particular voucher (in combination with *Cooperia* morphospecies and geographical origin). The *C*. *oncophora* and the Mexican *C*. *punctata* samples were obtained from different pools of larvae using isolates that have been characterized as single species isolates. Node support values represent results of the rapid bootstrapping analysis and of the Shimodaira-Hasegawa likelihood ration test before and behind the slash, respectively. Accession numbers for all new sequences are available from S2 Table.
(PDF)

S2 Fig Phylogenetic analysis using partial isotype 1 β-tubulin gene sequences. Sequences were aligned using M-Coffee and manually validated to ensure that codons were not disrupted by gaps. A phylogenetic tree was calculated using RAxML without partitioning the data. A sequence from *Haemonchus contortus was* included as outgroup. Samples were obtained from individual worms identified as *Cooperia pectinata* (*Cpec*, green), *Cooperia punctata* (*Cpun*, magenta), *Cooperia spatulata* (*Cspa*, red) and *Cooperia oncophora* (*Conc*, blue). Sequences derived from Brazil (B) and Mexcico (M) are indicated together with numbers indicating the particular voucher (in combination with *Cooperia* morphospecies and geographical origin). The *C*. *oncophora* and the Mexican *C*. *punctata* samples were obtained from different pools of larvae using isolates that have been characterized as single species isolates. Node support values represent results of the rapid bootstrapping analysis and of the Shimodaira-Hasegawa likelihood ration test before and behind the slash, respectively. Accession numbers for all new sequences are available from S2 Table.
(PDF)

S3 Fig Phylogenetic analysis using mitochondrial 12S rRNA sequences. Sequences were aligned using MAFFT and a phylogenetic tree was calculated using RAxML without partitioning the data. Sequences from *Teladorsagia circumcincta*, *Trichostrongylus vitrinus*, *Trichostrongylus axei*, *Haemonchus contortus* and *Haemonchus placei* were included as outgroups. Samples were obtained from individual worms identified as *Cooperia pectinata* (*Cpec*, green), *Cooperia punctata* (*Cpun*, magenta), *Cooperia spatulata* (*Cspa*, red) and *Cooperia oncophora* (*Conc*, blue). Sequences derived from Brazil (B) and Mexcico (M) are indicated together with numbers indicating the particular voucher (in combination with *Cooperia* morphospecies and geographical origin. The *C*. *oncophora* and the Mexican *C*. *punctata* samples were obtained from different pools of larvae using isolates that have been characterized as single species isolates. Node support values represent results of the rapid bootstrapping analysis and of the Shimodaira-Hasegawa likelihood ration test before and behind the slash, respectively. Accession numbers for all new sequences are available from S2 Table.
(PDF)

S4 Fig Phylogenetic analysis using partial *cox-2* sequences. Sequences were aligned using M-Coffee data were manually inspected to ensure that gaps did not disrupt codons. A phylogenetic tree was calculated using RAxML with separate partitions for codon positions 1 and 2 and codon position 3. Sequences from *Teladorsagia circumcincta*, *Trichostrongylus vitrinus*, *Trichostrongylus axei*, *Haemonchus contortus* and *Haemonchus placei* were included as outgroups. Samples were obtained from individual worms identified as *Cooperia pectinata* (*Cpec*, green), *Cooperia punctata* (*Cpun*, magenta), *Cooperia spatulata* (*Cspa*, red) and *Cooperia oncophora* (*Conc*, blue). Sequences derived from Brazil (B) and Mexcico (M) are indicated together with numbers indicating the particular voucher (in combination with *Cooperia* morphospecies and geographical origin. The *C*. *oncophora* and the Mexican *C*. *punctata* samples were obtained from different pools of larvae using isolates that have been characterized as single species isolates. Node support values represent results of the rapid bootstrapping analysis and of the Shimodaira-Hasegawa likelihood ration test before and behind the slash, respectively. Accession numbers for all new sequences are available from S2 Table.
(PDF)
