# Supplementary material for: Molecular marker sequences of cattle Cooperia species identify Cooperia spatulata as a morphotype of Cooperia punctata
Source: PLoS One. 2018 Jul 6;13(7):e0200390. doi: 10.1371/journal.pone.0200390 (PMC6034896; doi:10.1371/journal.pone.0200390)
Supplement: S1 Table — (PDF) [file pone.0200390.s001.pdf]

S1 Table

Primer sequences and PCR conditions

Target gene	Primer name	Sequence 5’ -> 3’	T_a[a] (°C)	Et[b] (s.)	Size (bp)[c]	References
12S gene	Coop12S_for_kurz	GTATAATACTTGTTCCAGAATAATC	51	20	~ 223	Designed in this study
	Coop12S_rev_kurz	CTTACAGTTTAAATACAACTTTAC				Designed in this study
Cytochrome oxidase II	COII_deg_for	ATKGARTAYCARTTTGGIGARTT	47	30	~ 452	Designed in this study
	COII_deg_rev	CTRTGRTTIGCICCRCARATYTC				Designed in this study
Internal transcribed spacer [d]	ITS1_part_for	TAGGTGAAACCTGCAGATGGAT	52	45	~ 1223	Modified from Demeler et al, 2013
	NC2	TTAGTTTCTTTTCCTCCGCT				Gasser et al, 1993
Partiall ß-tubulin isotype 1	CoPCR167fw	TATGGGCACTTTGCTTATTTCA	52	20	~ 267	Demeler et al., 2013
	CoPCR198+200rev	CCGGACATYGTGACAGACACTAGG				Modified from.Demeler et al., 2013
Sequencing primer	5.8S Seq_for	GCAGACGCTTAGAGTGGTG				Designed in this study
	5.8S Seq_rev	CAGACGTGCCGAAGGGAAAAC				Designed in this study

[a]Annealing temperature,

[b]Extention time

[c]Fragmentsize including Primer

[d]partiell internal transcribed spacer-1, 5.8S rRNA and complete internal transcribed spacer-2
