# Supplementary material for: Molecular marker sequences of cattle Cooperia species identify Cooperia spatulata as a morphotype of Cooperia punctata
Source: PLoS One. 2018 Jul 6;13(7):e0200390. doi: 10.1371/journal.pone.0200390 (PMC6034896; doi:10.1371/journal.pone.0200390)
Supplement: S2 Table — (PDF) [file pone.0200390.s002.pdf]

S2 Table. GenBank accession numbers for all sequences included in the phylogenetic analyses and not provin the figures.

Morphospecies	Country (Isolate)	Isolate/Voucher	12S rRNA	ITS-1/ITS-2	Cytochrome oxidase II	β-tubulin isotype 1
C. oncophora	UK	Weybridge C1	MH270705	MH267770	MH290287	MH290336
C. oncophora	UK	Weybridge C2	MH270704	MH267771	MH290288	MH290335
C. oncophora	UK	IVMres C3	MH270703	MH267772	MH290289	MH290334
C. oncophora	UK	IVMres C4	MH270702	MH267773	MH290290	MH290333
C. oncophora	NZ	NZres C5	MH270701	MH267774	MH290291	MH290332
C. pectinata	Brazil	P21	MH270690	MH267780	MH290292	MH290326
C. pectinata	Brazil	P22	MH270689	MH267781	MH290293	MH290325
C. pectinata	Brazil	P23	MH270688	MH267782	MH290294	MH290324
C. pectinata	Brazil	P24	n.a.	MH267783	MH290295	MH290323
C. pectinata	Brazil	P25	MH270687	MH267784	MH290296	MH290322
C. pectinata	Brazil	P31	MH270686	MH267785	n.a.	MH290321
C. pectinata	Brazil	P32	MH270685	n.a.	n.a.	MH290320
C. pectinata	Brazil	P33	MH270684	n.a.	MH290297	MH290319
C. punctata	Mexico	M1	MH270700	n.a.	n.a.	n.a.
C. punctata	Mexico	M11	MH270699	MH267775	MH290298	MH290331
C. punctata	Mexico	M12	MH270698	MH267776	MH290299	MH290330
C. punctata	Mexico	M13	MH270697	MH267777	MH290300	MH290329
C. punctata	Mexico	M14	MH270696	MH267778	MH290301	MH290328
C. punctata	Mexico	M15	MH270695	MH267779	MH290302	MH290327
C. punctata	Mexico	M2	MH270694	n.a.	n.a.	n.a.
C. punctata	Mexico	M3	MH270693	n.a.	n.a.	n.a.
C. punctata	Mexico	M4	MH270692	n.a.	n.a.	n.a.
C. punctata	Mexico	M5	MH270691	n.a.	n.a.	n.a.
C. punctata	Brazil	B21	MH270711	MH267765	MH290305	MH290342
C. punctata	Brazil	B22	MH270710	MH267766	n.a.	MH290341
C. punctata	Brazil	B23	n.a.	n.a.	MH290306	MH290340
C. punctata	Brazil	B24	MH270709	MH267767	MH290307	MH290339
C. punctata	Brazil	B25	MH270708	MH267768	MH290303	MH290338
C. punctata	Brazil	B31	MH270706	n.a.	n.a.	n.a.
C. punctata	Brazil	B33	MH270707	MH267769	MH290304	MH290337
C. spatulata	Brazil	S21	MH270683	MH267786	MH290308	MH290318
C. spatulata	Brazil	S22	MH270682	MH267787	MH290309	MH290317
C. spatulata	Brazil	S23	MH270681	MH267788	MH290311	MH290316
C. spatulata	Brazil	S24	MH270680	MH267789	MH290310	MH290315
C. spatulata	Brazil	S31	MH270678	MH267790	MH290312	MH290314
C. spatulata	Brazil	S31	n.a.	n.a.	MH290313	n.a.
C. spatulata	Brazil	S32	MH270679	MH267791	n.a.	n.a.

n.a., not available.
UK, United Kingdom
NZ, New Zealand
