# Supplementary figures and images for: Molecular marker sequences of cattle Cooperia species identify Cooperia spatulata as a morphotype of Cooperia punctata
Source: PLoS One. 2018 Jul 6;13(7):e0200390. doi: 10.1371/journal.pone.0200390 (PMC6034896; doi:10.1371/journal.pone.0200390)

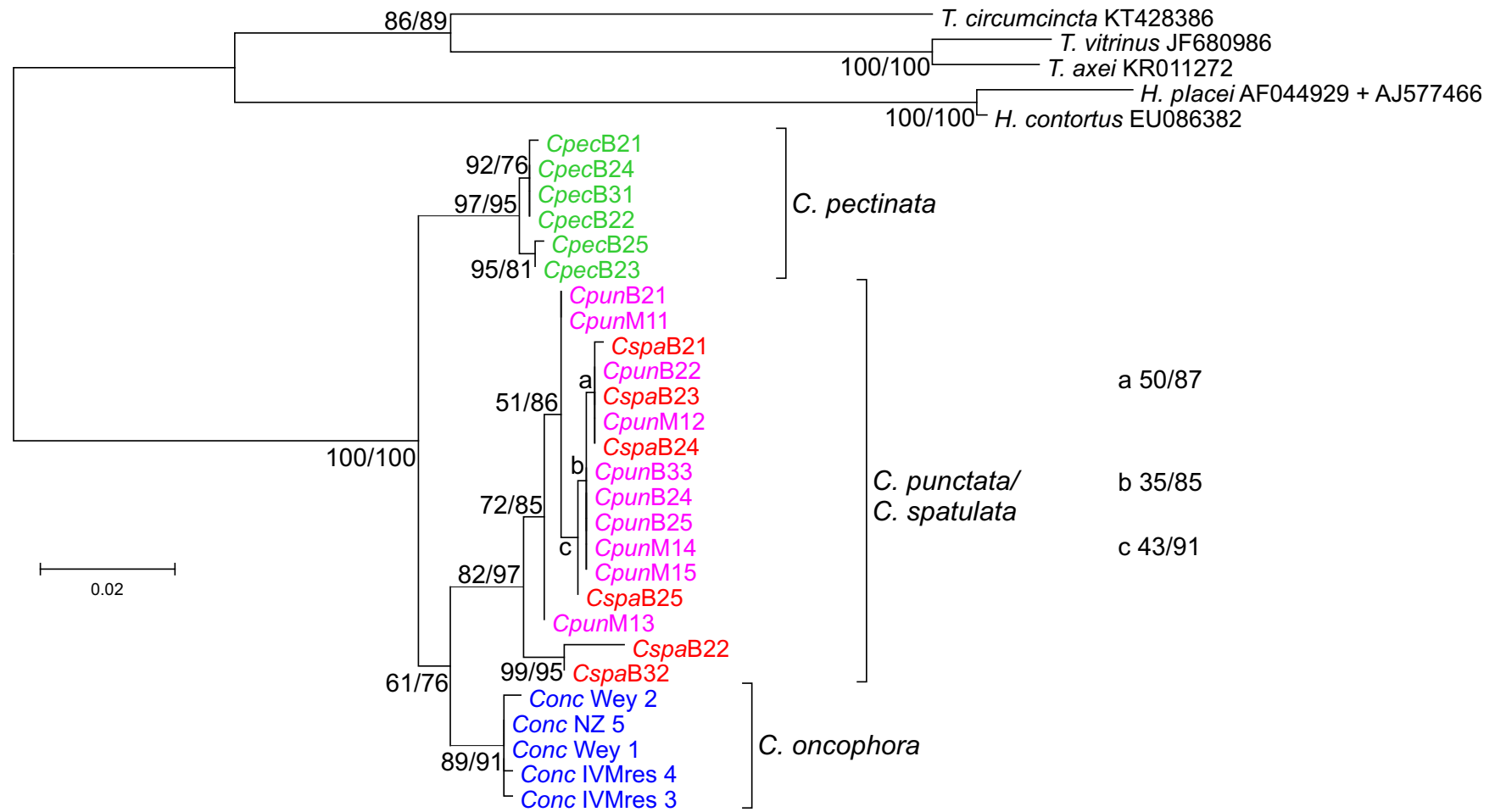

Supplement: S1 Fig — Sequences were aligned using MAFFT and a phylogenetic tree was calculated using RAxML without partitioning the data. Sequences from Teladorsagia circumcincta, Trichostrongylus vitrinus, Trichostrongylus axei, Haemonchus contortus and Haemonchus placei were included as outgroups. Samples were obtained from individual worms identified as Cooperia pectinata (Cpec, green), Cooperia punctata (Cpun, magenta), Cooperia spatulata (Cspa, red) and Cooperia oncophora (Conc, blue). Sequences derived from Brazil (B) and Mexcico (M) are indicated together with numbers indicating the particular voucher (in combination with Cooperia morphospecies and geographical origin). The C. oncophora and the Mexican C. punctata samples were obtained from different pools of larvae using isolates that have been characterized as single species isolates. Node support values represent results of the rapid bootstrapping analysis and of the Shimodaira-Hasegawa likelihood ration test before and behind the slash, respectively. Accession numbers for all new sequences are available from S2 Table. (PDF) [file pone.0200390.s003.pdf]

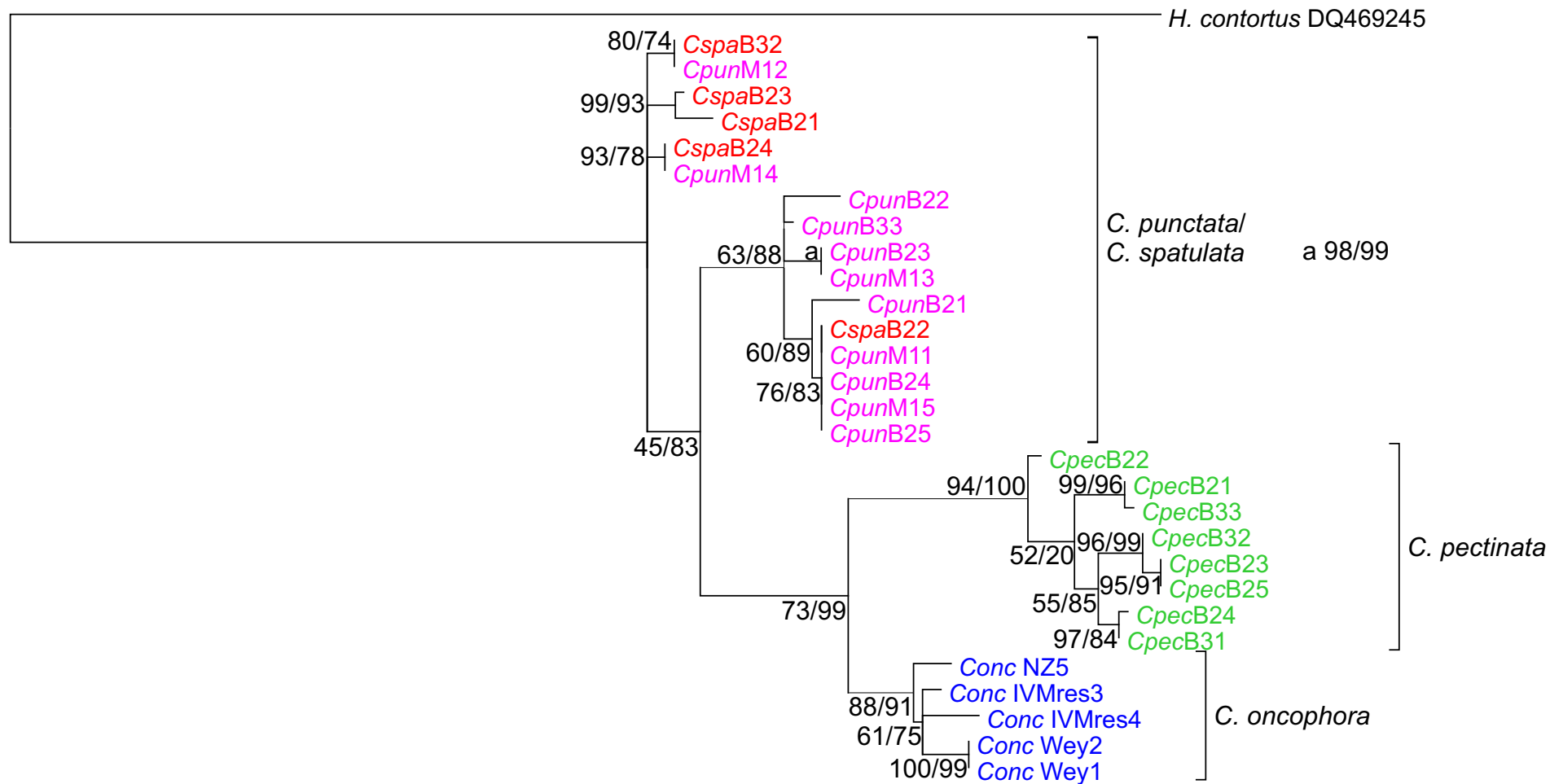

0.01

Supplement: S2 Fig — Sequences were aligned using M-Coffee and manually validated to ensure that codons were not disrupted by gaps. A phylogenetic tree was calculated using RAxML without partitioning the data. A sequence from Haemonchus contortus was included as outgroup. Samples were obtained from individual worms identified as Cooperia pectinata (Cpec, green), Cooperia punctata (Cpun, magenta), Cooperia spatulata (Cspa, red) and Cooperia oncophora (Conc, blue). Sequences derived from Brazil (B) and Mexcico (M) are indicated together with numbers indicating the particular voucher (in combination with Cooperia morphospecies and geographical origin). The C. oncophora and the Mexican C. punctata samples were obtained from different pools of larvae using isolates that have been characterized as single species isolates. Node support values represent results of the rapid bootstrapping analysis and of the Shimodaira-Hasegawa likelihood ration test before and behind the slash, respectively. Accession numbers for all new sequences are available from S2 Table. (PDF) [file pone.0200390.s004.pdf]

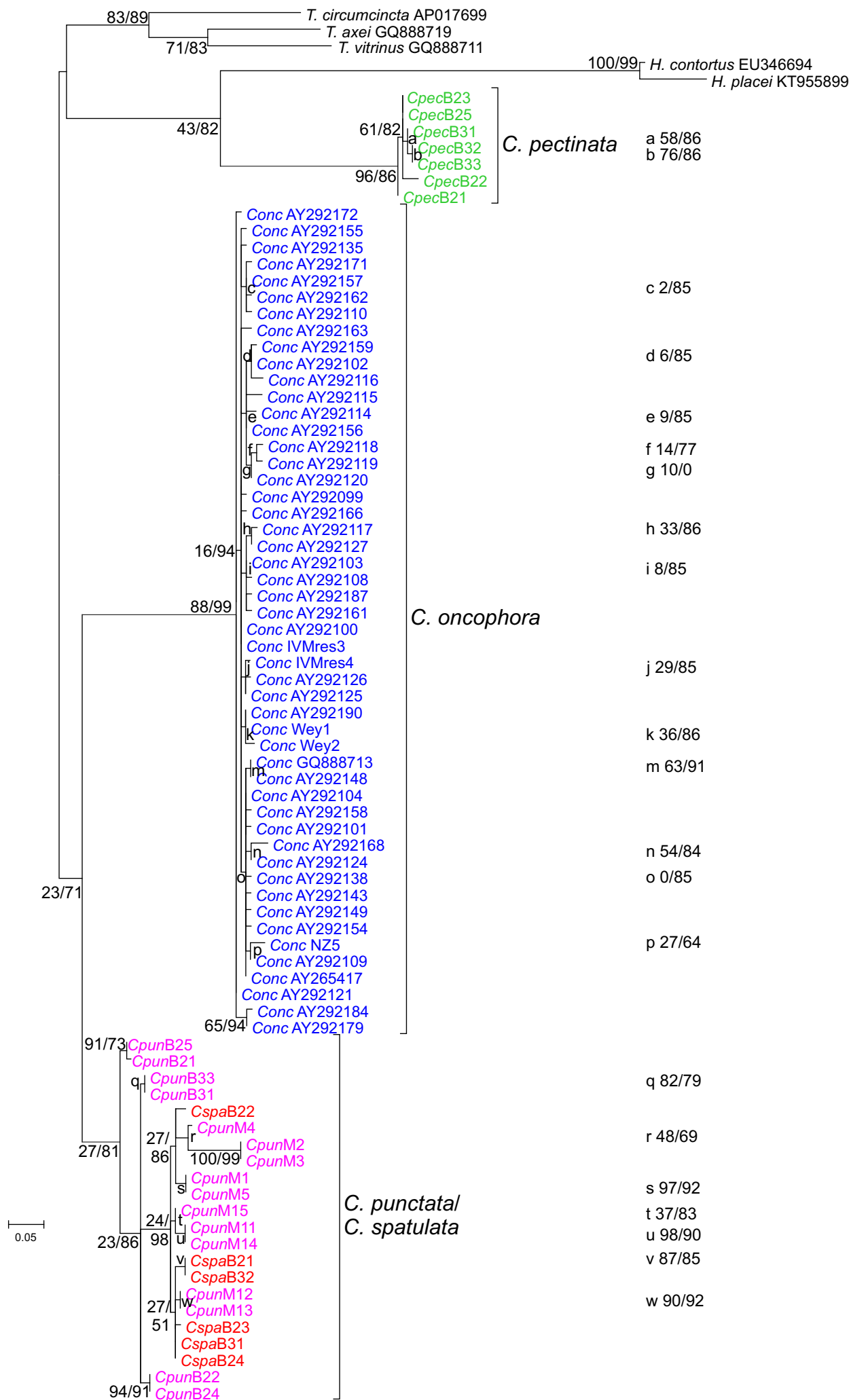

Supplement: S3 Fig — Sequences were aligned using MAFFT and a phylogenetic tree was calculated using RAxML without partitioning the data. Sequences from Teladorsagia circumcincta, Trichostrongylus vitrinus, Trichostrongylus axei, Haemonchus contortus and Haemonchus placei were included as outgroups. Samples were obtained from individual worms identified as Cooperia pectinata (Cpec, green), Cooperia punctata (Cpun, magenta), Cooperia spatulata (Cspa, red) and Cooperia oncophora (Conc, blue). Sequences derived from Brazil (B) and Mexcico (M) are indicated together with numbers indicating the particular voucher (in combination with Cooperia morphospecies and geographical origin. The C. oncophora and the Mexican C. punctata samples were obtained from different pools of larvae using isolates that have been characterized as single species isolates. Node support values represent results of the rapid bootstrapping analysis and of the Shimodaira-Hasegawa likelihood ration test before and behind the slash, respectively. Accession numbers for all new sequences are available from S2 Table. (PDF) [file pone.0200390.s005.pdf]

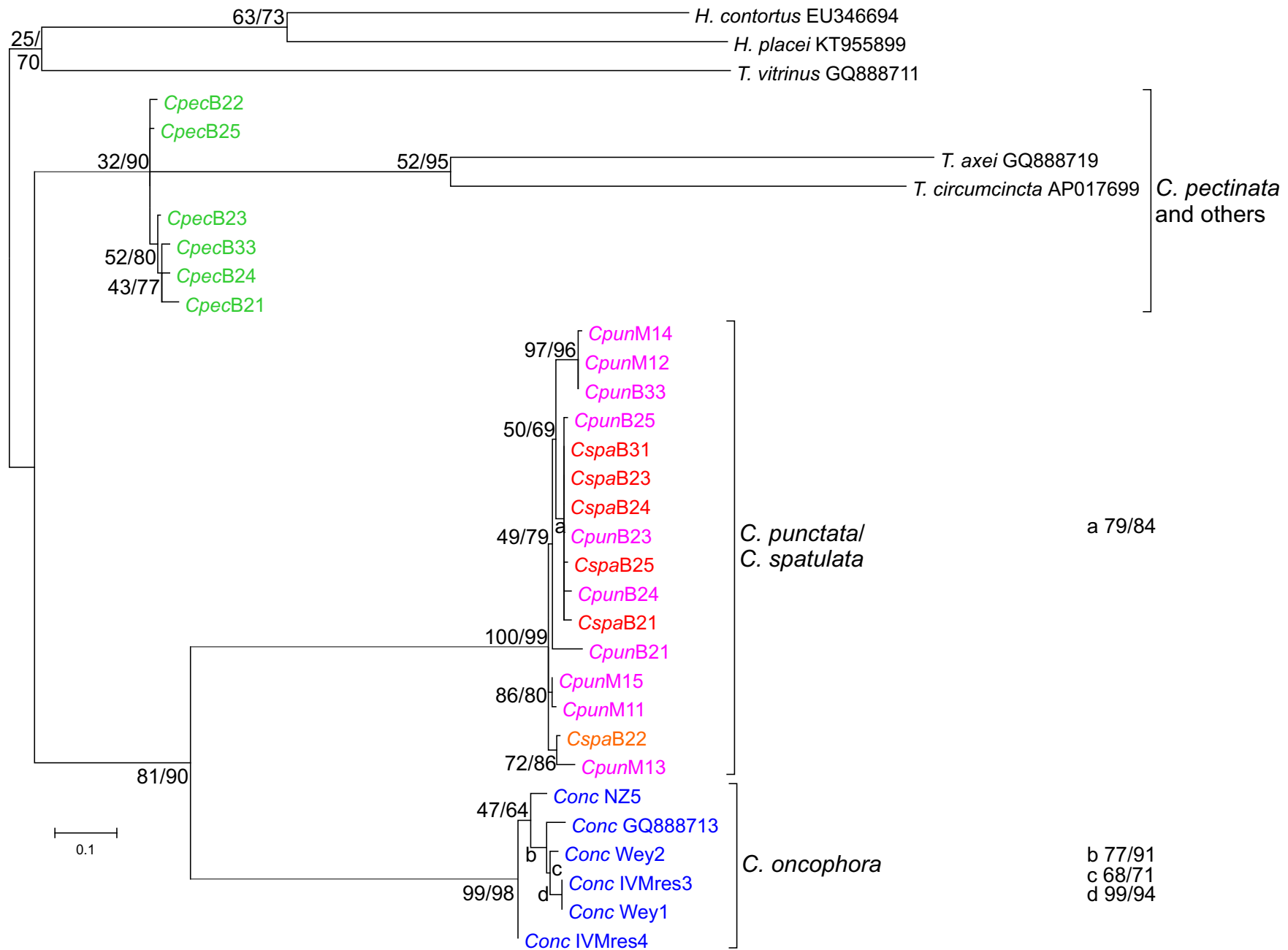

Supplement: S4 Fig — Sequences were aligned using M-Coffee data were manually inspected to ensure that gaps did not disrupt codons. A phylogenetic tree was calculated using RAxML with separate partitions for codon positions 1 and 2 and codon position 3. Sequences from Teladorsagia circumcincta, Trichostrongylus vitrinus, Trichostrongylus axei, Haemonchus contortus and Haemonchus placei were included as outgroups. Samples were obtained from individual worms identified as Cooperia pectinata (Cpec, green), Cooperia punctata (Cpun, magenta), Cooperia spatulata (Cspa, red) and Cooperia oncophora (Conc, blue). Sequences derived from Brazil (B) and Mexcico (M) are indicated together with numbers indicating the particular voucher (in combination with Cooperia morphospecies and geographical origin. The C. oncophora and the Mexican C. punctata samples were obtained from different pools of larvae using isolates that have been characterized as single species isolates. Node support values represent results of the rapid bootstrapping analysis and of the Shimodaira-Hasegawa likelihood ration test before and behind the slash, respectively. Accession numbers for all new sequences are available from S2 Table. (PDF) [file pone.0200390.s006.pdf]
